# Quick change: post-transcriptional regulation in *Pseudomonas*

**DOI:** 10.1093/femsle/fnx125

**Published:** 2017-06-12

**Authors:** Lucia Grenga, Richard H. Little, Jacob G. Malone

**Affiliations:** 1 John Innes Centre, Norwich Research Park, Colney Lane, Norwich NR4 7UH, UK; 2 University of East Anglia, Norwich Research Park, Norwich, NR4 7TJ, UK

**Keywords:** translational control, *Pseudomonas*, post-transcriptional regulation, regulatory responses, signalling pathway, ribosomal modification

## Abstract

*Pseudomonas* species have evolved dynamic and intricate regulatory networks to fine-tune gene expression, with complex regulation occurring at every stage in the processing of genetic information. This approach enables *Pseudomonas* to generate precise individual responses to the environment in order to improve their fitness and resource economy. The weak correlations we observe between RNA and protein abundance highlight the significant regulatory contribution of a series of intersecting post-transcriptional pathways, influencing mRNA stability, translational activity and ribosome function, to *Pseudomonas* environmental responses. This review examines our current understanding of three major post-transcriptional regulatory systems in *Pseudomonas* spp.; Gac/Rsm, Hfq and RimK, and presents an overview of new research frontiers, emerging genome-wide methodologies, and their potential for the study of global regulatory responses in *Pseudomonas*.

## Teaser

*Pseudomonas* species have evolved dynamic and intricate regulatory networks to quickly fine-tune gene expression in response to environmental stimuli.

## POST-TRANSCRIPTIONAL REGULATORY MECHANISMS

**Figure 1.**
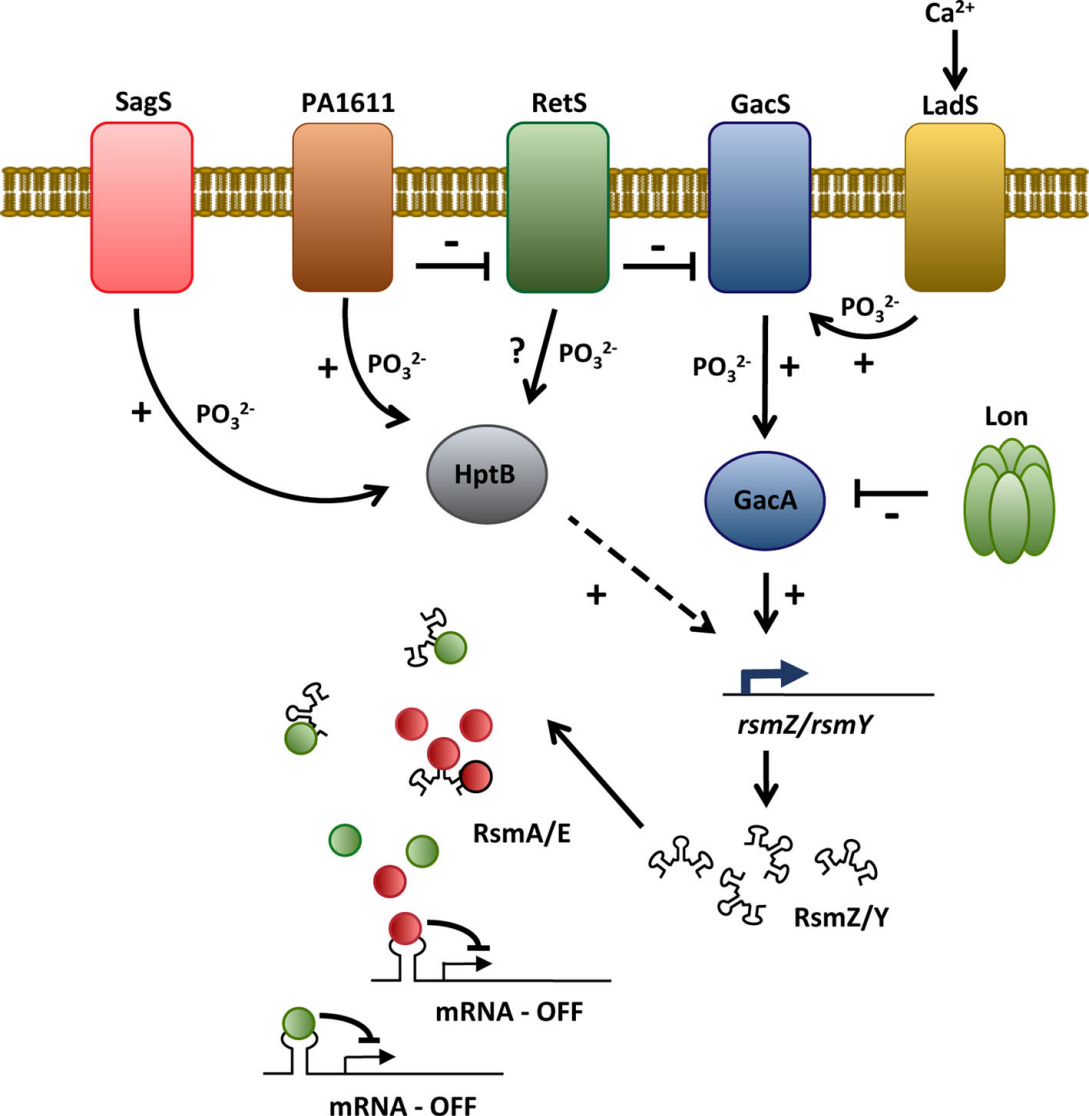
The Gac/Rsm regulatory network in *P. aeruginosa*. An integrated response from multiple membrane-bound histidine kinases controls the activity of the response regulator GacA, which in turn controls expression of the RsmZ/Y sRNAs. These sRNA molecules inhibit the translational regulatory proteins RsmA and RsmE (red and green circles), leading to altered translation of their target mRNAs. Other proteins that influence Gac/Rsm function include the phosphotransfer protein HptB and the Lon protease complex.

One of the most well-understood pathways responsible for integrating external stimuli into post-transcriptional control in *Pseudomonas* is the Gac/Rsm signalling pathway (Coggan and Wolfgang 2012). Gac/Rsm is a widespread system that controls biofilm formation, virulence, motility and external stress responses in many different bacterial species (Brencic and Lory 2009; Chambers and Sauer 2013), and represents a major determinant of the switch between chronic and acute lifestyles in *Pseudomonas aeruginosa*. While many of the core network components and their functions in the signalling cascade have been described in detail (Brencic *et al.*^2009^; Goodman *et al.*^2009^) (Fig. 1), in recent years Gac/Rsm has also been shown to regulate several downstream signalling pathways including transcriptional regulators, quorum sensing and the second messenger cyclic-di-GMP (Brencic and Lory 2009; Chambers and Sauer 2013), markedly increasing the complexity of the system.

At the heart of the Gac/Rsm pathway are the small RNA molecules RsmY and RsmZ. The abundance of these sRNAs ultimately dictates the output of the Gac/Rsm system, and as such their transcription is subject to tight and complex regulation by the GacAS two-component signalling system. GacS is a transmembrane histidine protein kinase (HPK), and activates its cognate response regulator GacA by phosphotransfer (Goodman *et al.*^2009^). Upon phosphorylation, GacA promotes transcription of RsmY/Z (Brencic *et al.*^2009^), which contain multiple GGA trinucleotides in exposed stem-loops of their predicted secondary structures (Schubert *et al.*^2007^; Lapouge *et al.*^2013^). RsmA and the related protein RsmE (Reimmann *et al.*^2005^) are small (7 kDa) proteins that specifically recognise and bind to conserved GGA sequences in the 5΄ leader regions of target mRNAs. RsmA/RsmE binding affects mRNA stability, and/or prevents interactions between the 30S ribosomal subunit and the ribosomal binding site, thus inhibiting translation initiation (Heurlier *et al.*^2004^; Reimmann *et al.*^2005^). RsmA/E activity is in turn inhibited by RsmY/Z, which titrate RsmA/E away from the 5΄ mRNA leader sequences in their target mRNAs (Heurlier *et al.*^2004^) (Fig. 1). The relationship between *Pseudomonas fluorescens* RsmE and RsmZ has recently been defined at the molecular level, with RsmE protein dimers assembling sequentially onto the RsmZ sRNA within a narrow affinity range (100–200 nM *K*_d_ in *P. fluorescens*), and showing positive binding cooperativity (Duss *et al.*^2014^). The GacAS system is itself controlled by three additional HPK hybrid proteins: RetS, PA1611 and LadS (Ventre *et al.*^2006^; Kong *et al.*^2013^) (Fig. 1). These HPKs are present in most pseudomonads, although the regulatory network can vary between individual species (Chatterjee *et al.*^2003^; Wei *et al.*^2013^). In *P. aeruginosa*, RetS functions as an antagonist of GacS, and suppresses RsmZ/Y levels (Goodman *et al.*^2004^). However, rather than operating via a conventional HPK phosphotransfer mechanism, RetS binds to and inhibits GacS, blocking its autophosphorylation and preventing the downstream phosphorylation of GacA (Goodman *et al.*^2009^). Conversely, PA1611 interacts directly with RetS in *P. aeruginosa*, thus enabling the activation of GacS (Kong *et al.*^2013^; Bhagirath *et al.*^2017^). LadS positively controls *rsmY*/*Z* expression through a phosphorelay resulting in phosphotransfer to the Histidine phosphotransfer (HPT) domain of GacS (Chambonnier *et al.*^2016^). In *P. aeruginosa*, although interestingly not in other tested *Pseudomonas* species, LadS activation occurs following calcium binding to its periplasmic DISMED2 domain, which activates its kinase activity (Broder, Jaeger and Jenal 2016) (Fig. 1).

Several additional signalling proteins, sRNAs and other pathways are implicated in the control of Gac/Rsm (Chambers and Sauer 2013). For example, BswR, an XRE-type transcriptional regulator in *P. aeruginosa*, controls *rsmZ* transcription (Wang *et al.*^2014^). The histidine phosphotransfer protein HptB indirectly controls *rsmY* expression under planktonic growth conditions. HptB is the phosphorylation target of four HPKs, including RetS, PA1611, PA1976 and SagS (Lin *et al.*^2006^; Hsu *et al.*^2008^). SagS also controls the Biofilm Initiation two-component system BfiSR, a key regulator of the initial stages of biofilm formation, and itself a repressor of *rsmZ* expression (Petrova and Sauer 2011). In addition to RsmY/RsmZ, other small RNAs can also influence RsmA/E function. In *P. aeruginosa*, the sRNA RsmW specifically binds to RsmA *in vitro*, restoring biofilm production and reducing swarming in an *rsmYZ* mutant. RsmW expression is elevated in late stationary versus logarithmic growth, and at higher temperatures (Miller *et al.*^2016^). RsmY and RsmZ are also differentially regulated by the conditions in the growth environment (Jean-Pierre, Tremblay and Deziel 2016). Finally, the ATP-dependent protease Lon negatively regulates the Gac/Rsm cascade, with *lon* mutants showing increased stability and steady-state levels of GacA in late exponential growth (Takeuchi *et al.*^2014^).

**Figure 2.**
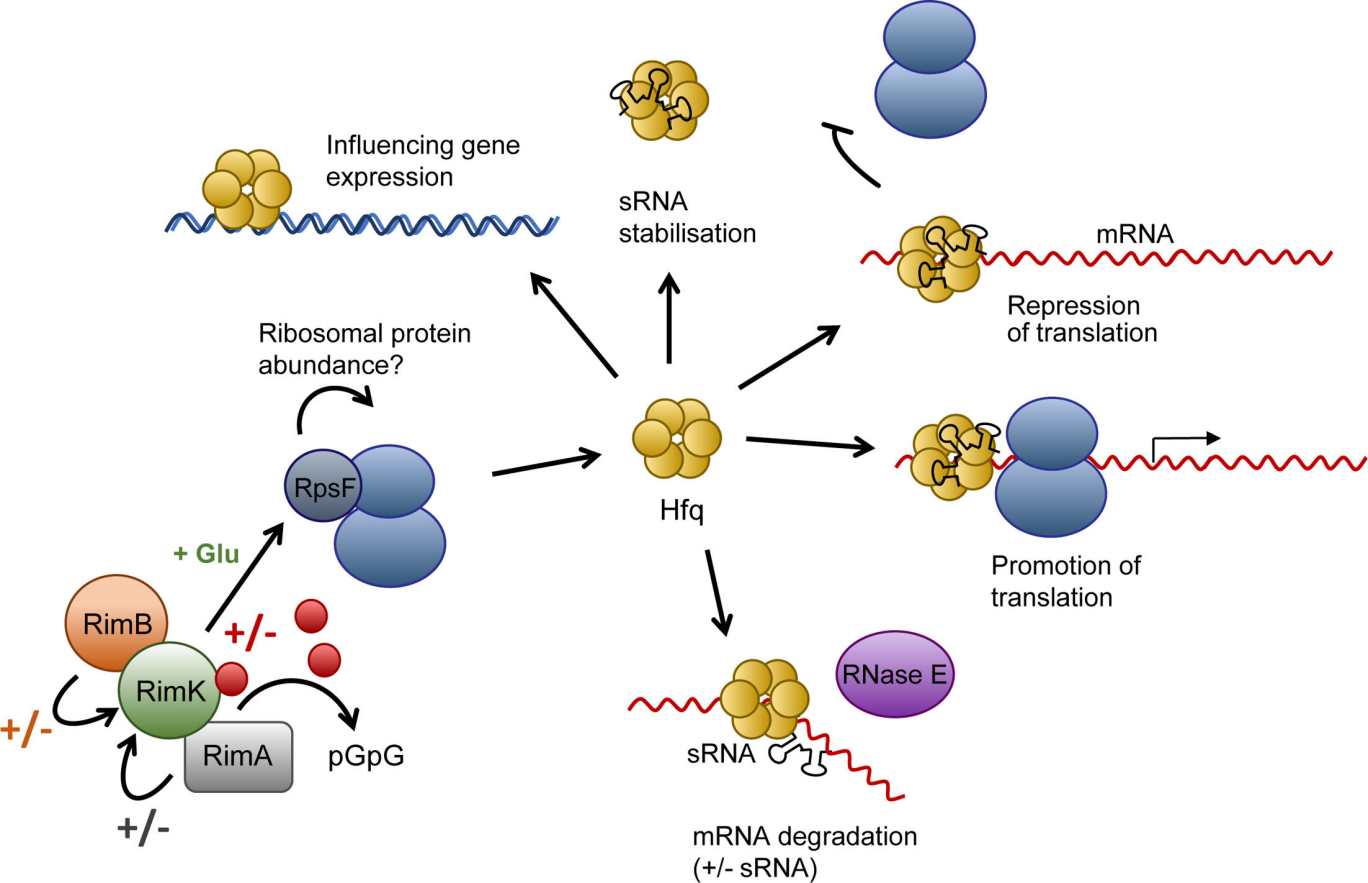
The Rim and Hfq regulatory networks in *Pseudomonas* spp. The RimK glutamate ligase sequentially adds glutamate residues to the C-terminus of ribosomal protein S6 (RpsF). RimK activity is tightly controlled through direct interaction with the second messenger cyclic-di-GMP (red circles), RimB and the cyclic-di-GMP phosphodiesterase RimA. RpsF glutamation affects ribosome function, which leads to altered Hfq abundance via an as-yet unidentified mechanism. Hfq is a pleiotropic regulator of mRNA/sRNA stability, mRNA translation and gene transcription. These processes are mediated through a diverse series of Hfq–RNA/DNA interactions.

The Gac/Rsm system shows extensive regulatory overlap with a second major post-transcriptional regulator; Hfq. Hfq is a small, hexameric RNA-binding protein with several discrete regulatory functions (Fig. 2) (Vogel and Luisi 2011). Hfq function is dictated in large part by the abundance of its various sRNA binding partners. Unlike RsmA/E, which has only two or three cognate sRNAs, Hfq binds to many different sRNA molecules that are expressed under different conditions (Vogel and Luisi 2011; Chambers and Sauer 2013). It functions as an RNA chaperone, facilitating binding between regulatory sRNAs and their mRNA targets (Moller *et al.*^2002^; Maki *et al.*^2008^). Hfq also targets the specific degradation of selected mRNAs (Moll *et al.*^2003^; Afonyushkin *et al.*^2005^; Morita, Maki and Aiba 2005) and can act as a direct repressor of mRNA translation (Desnoyers and Masse 2012). Hfq binding also acts to protect sRNAs from degradation by polynucleotide phosphorylase (PNPase) and other enzymes (Andrade *et al.*^2012^). Finally, it can regulate gene expression by influencing mRNA polyadenylation (Valentin-Hansen, Eriksen and Udesen 2004), or through direct interaction with DNA (Fig. 2) (Cech *et al.*^2016^). Hfq binds to and stabilises RsmY in *P. aeruginosa* (Sonnleitner *et al.*^2006^), while the RsmA homologue CsrA represses Hfq translation in *Escherichia coli* (Baker *et al.*^2007^). Furthermore, *E. coli* CsrA and Hfq share at least one regulatory sRNA (Jorgensen *et al.*^2013^). Similarly to GacA (Takeuchi *et al.*^2014^), Hfq levels increase in a *P. aeruginosa lon* mutant background (Fernandez *et al.*^2016^). Regulation of oxidative stress response proteins (Zhang *et al.*^1998^; Fields and Thompson 2008) and the Fis global transcriptional regulator (via the sRNA RgsA; Lu *et al.*^2016^) have also been linked to both Hfq and Gac/Rsm. This regulatory connection is reflected in the large number of shared phenotypes between *rsmA/E* and *hfq* mutants in *Pseudomonas* species, with disruption of either gene leading to increased surface attachment, reduced motility and disruption of virulence (Brencic and Lory 2009; Irie *et al.*^2010^; Little *et al.*^2016^).

Hfq controls a wide variety of phenotypes, with common regulatory targets emerging from studies of closely related bacteria. In *Pseudomonas* and other proteobacteria, Hfq controls carbon catabolite repression (Sonnleitner and Bläsi 2014), and negatively regulates both amino acid ABC transporters (Sonnleitner *et al.*^2006^; Gao *et al.*^2010^; Sobrero *et al.*^2012^; Little *et al.*^2016^), and pathways underpinning biofilm formation (Jorgensen *et al.*^2012^; Thomason *et al.*^2012^). Conversely, Hfq mRNA stabilisation exerts complex, but generally positive, effects on motility (Mulcahy *et al.*^2008^; Gao *et al.*^2010^) and virulence (Sonnleitner *et al.*^2003^). Hfq has also been implicated in the control of iron homeostasis (Sobrero *et al.*^2012^) and enables the environmental stress-tolerance super-phenotype in *Pseudomonas putida* (Arce-Rodriguez *et al.*^2016^). In *P. fluorescens*, Hfq plays an important role in niche adaptation, with reduced Hfq levels resulting in phenotypes including reduced motility, increased surface attachment, and compromised rhizosphere colonisation (Little *et al.*^2016^).

Hfq and its target sRNAs have been the subject of intensive research in several bacteria. As well as structural/biochemical studies of Hfq–RNA complexes (Mikulecky *et al.*^2004^; Link, Valentin-Hansen and Brennan 2009), a number of recent studies have examined the relationship between Hfq and RNA using global methods such as CLIP-Seq analysis to identify Hfq-bound RNAs (Sittka, Rolle and Vogel 2009; Holmqvist *et al.*^2016^) and transcriptional and proteomic surveys of *hfq* deletion mutants (Sonnleitner *et al.*^2006^; Gao *et al.*^2010^; Sobrero *et al.*^2012^; Boudry *et al.*^2014^). Global proteomic and transcriptomic analyses have been conducted for *hfq* mutants of *P. putida* (Arce-Rodriguez *et al.*^2016^) and *P. aeruginosa* (Sonnleitner *et al.*^2006^), respectively, and implicate Hfq in the control of pathways including acetoin and metabolism, ABC and MFS transporters, quorum sensing, and siderophore and phenazine production. These global analytical methods promise to greatly increase our mechanistic understanding of post-transcriptional regulation by the well-studied Gac/Rsm and Hfq pathways, and are discussed in more detail in the final section of this review.

## NOVEL MECHANISMS OF TRANSLATIONAL REGULATION

In addition to these well-studied pathways for post-transcriptional control, entirely new regulatory mechanisms are still being discovered. The specific alteration of ribosome function by post-translational modification of its associated proteins represents a significant, and to date largely unexplored, regulatory process (Little *et al.*^2016^). Fifty-seven proteins have been identified in the bacterial ribosome, many of which are essential, and 34 of which are universally conserved (Bubunenko, Baker and Court 2007). Intriguingly, multiple ribosomal proteins are subject to post-translational regulation by acetylation, methylation, methylthiolation, and the removal or addition of C-terminal amino acid residues. While the purpose of such modifications is in most cases still unknown (Nesterchuk, Sergiev and Dontsova 2011), their existence strongly suggests that aspects of ribosomal behaviour may be subject to dynamic regulation through a process of ribosomal specialisation. It is tempting to posit that changes to the ribosome will result in corresponding changes to the cellular proteome as a consequence of altered ribosome–mRNA recognition, changes to translational efficiency, or other post-transcriptional mechanisms. Until relatively recently this has been difficult to test, as technological limitations coupled with a lack of searchable peptide sequence databases have rendered quantitative characterisation of cellular proteomes difficult, if not impossible. Advances in liquid chromatography-coupled mass analysis, sample labelling methods (Unwin 2010), and a critical mass of genome sequence data have revolutionised the field of proteomics. A recent study by our laboratory (Little *et al.*^2016^) has exploited these advances to probe the consequences of a particular ribosomal modification, revealing unexpectedly large and specific alterations in the cellular proteome.

In this work, we examined the effects of post-translational modification of the ribosomal protein RpsF. RpsF is located in the central domain of the 30S ribosomal subunit, where it interacts with both the ribosomal RNA and the protein S18 (Agalarov *et al.*^2000^). RpsF is modified by RimK, a member of the ATP-dependent ATP-Grasp superfamily, by the addition of glutamate residues at its C-terminus (Kang *et al.*^1989^). This modification is associated with profound effects on the structure and function of the *Pseudomonas* ribosome. Quantitative Liquid chromatography–mass spectrometry (LC–MS/MS) analysis of labelled peptides revealed that *rimK* deletion leads to significantly lower abundance of multiple ribosomal proteins, alongside increased stress response, amino acid transport and metal iron-scavenging pathways. No significant alterations were detected in the levels of rRNA, or the mRNAs of differentially translated proteins in the *rimK* mutant, suggesting that RpsF modification specifically changes ribosome function in some way, and this leads to altered proteome composition.

In the mutualistic plant-growth-promoting rhizobacteria *P. fluorescens*, the *rimK*-encoding operon is highly upregulated during early stage colonisation of the rhizosphere, suggesting an important role for RimK function in this period (Little *et al.*^2016^). This transcriptional regulation is reinforced by the tight control exerted on RimK protein activity, both transcriptionally and through interactions with the other components of the Rim operon (RimA, RimB) and the signalling molecule cyclic-di-GMP. RimA/B and cyclic-di-GMP interact directly with the RimK enzyme and substantially influence its ATPase and glutamate ligase activities, although the mechanistic details of the signalling network are currently poorly defined (Fig. 2) (Little *et al.*^2016^). In any event, modification of RpsF correlates with a post-transcriptional output favouring a motile, virulent state. This fits with the observed increase in *rimK* expression seen during the early stages of plant root colonisation, when cells need to rapidly colonise the spatial environment of the rhizosphere. Conversely, lack of RpsF modification is associated with protein changes that prioritise long-term rhizosphere adaptation, such as surface attachment, resource acquisition and stress resistance. In addition to controlling phenotypes associated with colonisation and metabolic adaptation, RimK also plays an important role in the virulence of both human and plant pathogenic pseudomonads (Little *et al.*^2016^).

A number of unanswered questions remain relating to the regulation and mechanism of action of the Rim pathway. Firstly, we do not yet fully understand how exactly RimK is controlled. How does the external environment influence RimK activity? What is the role of the widespread signalling molecule cyclic-di-GMP in RimK regulation? Related to this, how does control of RimK link into the wider network of post-transcriptional regulation in *Pseudomonas*? RsmA has a complex regulatory relationship with cyclic-di-GMP, both controlling its metabolism (Chambers and Sauer 2013) and subject to cyclic-di-GMP regulation itself (Moscoso *et al.*^2014^). This raises the possibility that RsmA and RimK may form part of a single, integrated pathway under the ultimate control of cyclic-di-GMP. A second major research area concerns the mechanistic function of RimK. How does RimK ribosomal modification lead to altered proteome composition? Is this a consequence of altered translation, or mRNA recognition by the modified ribosomes, or possibly a combination of both? Many of the proteomic changes producing Δ*rimK* phenotypes could be rationalised by the observed reduction in levels of the RNA-binding post-transcriptional regulator Hfq (Little *et al.*^2016^). Thus, it is important to determine the extent to which Rim tunes the proteome by controlling Hfq levels, and exactly how this control takes place.

The determination of RimK function highlights an intriguing new mechanism for post-transcriptional control that links changes in ribosome function, and hence proteome composition, to the dynamic, controlled modification of ribosomal proteins (Little *et al.*^2016^). In turn, this finding raises major implications for studies of other ribosomal modifications, several of which may also represent novel post-translational regulatory systems. If this turns out to be the case, it will further transform our understanding of post-transcriptional regulation in bacteria. In the final section of this review, we will discuss some of the emerging genome-wide methodologies that are allowing researchers to examine new aspects of post-transcriptional regulation in bacteria, and may give us answers to the outstanding questions raised above.

## EMERGING GENOME-WIDE METHODOLOGIES FOR INVESTIGATING TRANSLATIONAL REGULATION

**Figure 3.**
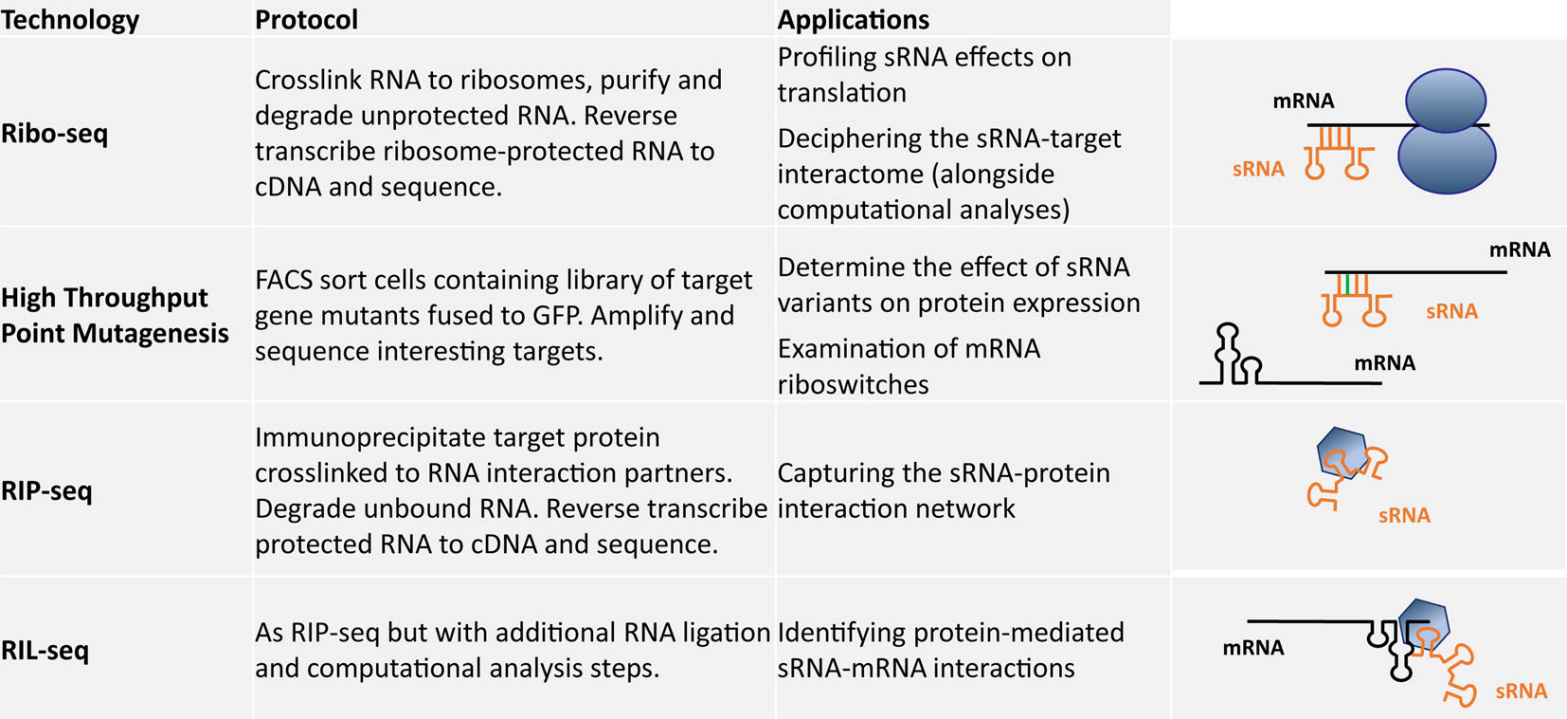
Emerging genome-wide methodologies. Overview of the new technologies developed to study mechanisms of translational regulation to a finer resolution. The subject, methodology and range of applications for each technique are summarised in each case.

While advances in quantitative proteomics enabled us to examine the impact of RimK on the *Pseudomonas* proteome, the development of additional, novel technologies are expanding our ability to probe other important mechanisms of translational regulation to a finer resolution than has previously been possible (Fig. 3).

Translational regulation of gene expression is a ribonucleoprotein-driven process, which involves both non-coding RNAs and RNA binding proteins (RBPs). A large complement of non-coding RNAs affect gene expression by employing multiple distinct regulatory mechanisms, at the level of translation initiation by modulating ribosome recruitment, and/or at the level of transcript abundance by modulating transcript degradation (Barquist and Vogel 2015). Deciphering the sRNA–target interactome is an essential step toward understanding the roles of sRNA in the cellular network. However, computational identification of sRNA targets can be challenging. sRNA–mRNA hybridisation is frequently influenced by sRNA secondary structure, and base-paired regions between RNAs are generally short and can include multiple discontinuous stretches of sequence (Wang *et al.*^2015^). To identify the regulatory targets of RyhB, one of the best-studied sRNAs in *Escherichia coli*, at the genome level Wang *et al.* established ribosome-profiling experiments (Ribo-seq) in bacteria (Fig. 3). Ribo-seq is a state-of-the-art technology that enables comprehensive and quantitative measurements of translation. Like many recent high-throughput techniques, it adapts an established technology to take advantage of the massively parallel measurements afforded by modern short-read sequencing. In the case of Ribo-seq, ribosomes bound to actively translated mRNAs are purified from cell lysates. Following digestion of the unprotected RNA fraction, the protected, ribosome-bound RNA is reverse transcribed to cDNA and sequenced. By identifying the precise positions of ribosomes on the transcript, ribosomal profiling experiments have unveiled key insights into the composition and regulation of the expressed proteome (Ingolia 2016). Ribo-seq is a powerful approach for the experimental identification of sRNA targets, and can reveal sRNA regulation both at the level of mRNA stability and at the translational level. However, while Ribo-seq can identify target mRNAs, it cannot reveal precise sites of sRNA:target hybridisation. Moving forward, sRNA target prediction algorithms could be combined with Ribo-seq datasets to facilitate guided target site identification, where predictions are focused on a subset of mRNAs rather than the whole transcriptome.

Many bacterial sRNAs are at least partially dependent on RBPs, such as the previously introduced RNA chaperone Hfq, for their function (Van Assche *et al.*^2015^). Approaches combining *in vivo* crosslinking and RNA deep sequencing have been increasingly used to globally map the cellular RNA ligands and binding sites of RBPs *in vivo* (Holmqvist *et al.*^2016^). Recent approaches include a UV crosslinking step, which offers several advantages over traditional co-immunoprecipitation (Zhang and Darnell 2011). These large-scale methods provide a global view of the RNA molecules bound to individual RBPs, although specific sRNA–target pairs can only be indirectly deduced by additional, sequence-dependent predictive schemes. To overcome this limitation, Melamed and colleagues (Melamed *et al.*^2016^) developed a broadly applicable methodology termed RIL-seq (RNA interaction by ligation and sequencing, Fig. 3). RIL-seq incorporates an additional RNA ligation step into the workflow of a conventional RNA pull-down experiment to create sRNA-mRNA chimeric fragments, followed by advanced computational analysis of the resulting cDNA library to identify interacting RNA pairs from the dataset of protein interaction partners. Applied to the *in vivo* transcriptome-wide identification of interactions involving Hfq-associated sRNA, this technique enabled the discovery of dynamic changes in the Hfq-mediated sRNA interactome with changing cellular conditions (Melamed *et al.*^2016^).

Integral features of individual mRNAs can also influence translation efficiency, and in many cases are directly involved in altering gene expression in response to changing cellular conditions or environmental stimuli (Meyer 2017). Specific motifs in the 5΄ untranslated region (UTR) of certain mRNAs can regulate gene expression in response to temperature, metals and small metabolite ligands. Such structures, known as riboswitches regulate metabolism and virulence by altering mRNA secondary structure to block ribosome access or induce early transcription termination (Fang *et al.*^2016^). In addition to this role, riboswitches are also involved in the regulation of non-coding RNA expression, representing a novel mechanism of signal integration in bacteria. In both cases, high-throughput point mutagenesis has enabled the identification of functional post-transcriptional regulatory elements. This method uses fluorescence-activated cell sorting (FACS) to categorise cells containing a mutant library based on the gene of interest fused to green fluorescent protein (GFP). This enables researchers to associate all possible mutations (including synonymous single-nucleotide polymorphisms (SNPs) that induce structural changes in the transcribed RNA) in a selected sequence with changes in gene expression (Holmqvist, Reimegård and Wagner 2013).

The plasticity of bacterial regulatory networks confers both versatility and efficiency, as multiple signals can be integrated to control the expression of common responses. To probe the intersecting contributions of the various inputs to bacterial gene expression, future analyses of post-transcriptional regulation are likely to involve the integration of several omics methods to produce comprehensive models for bacterial adaptation to external challenges. A recent demonstration of this approach compared relative changes in total mRNA with translational changes (polysome fractions) and protein abundance to provide a comprehensive study of bacterial stress responses in *Rhodobacter sphaeroides* (Berghoff *et al.*^2013^).

## CONCLUDING REMARKS

Despite the insights we have gained to date, the list of unresolved questions within the field of *Pseudomonas* post-transcriptional regulation remains very long. Many more RNA regulators are likely to be discovered, alongside novel regulatory mechanisms and refinements of existing pathways. Recent advancements in high throughput sequencing and bioinformatics, combined with novel approaches including quantitative proteomics, Ribo-seq, RIL-seq and various other omics techniques (Schulmeyer and Yahr 2017) present significant opportunities to discover and define exciting new mechanisms of post-transcriptional control.

***Conflict of interest.*** None declared.
